# The influence of complex and threatening environments in early life on brain size and behaviour

**DOI:** 10.1098/rspb.2015.2564

**Published:** 2016-01-27

**Authors:** C. DePasquale, T. Neuberger, A. M. Hirrlinger, V. A. Braithwaite

**Affiliations:** 1 Department of Biology, Pennsylvania State University—Altoona, Altoona, PA, USA; 2 Center for Brain, Behavior, and Cognition, Pennsylvania State University, University Park, PA, USA; 3 Department of Ecosystem Science and Management, Pennsylvania State University, University Park, PA, USA; 4 Huck Institutes of the Life Sciences, Pennsylvania State University, University Park, PA, USA; 5 Department of Bioengineering, Pennsylvania State University, University Park, PA, USA; 6 Department of Biology, Pennsylvania State University, University Park, PA, USA

**Keywords:** environmental enrichment, zebrafish (*Danio rerio*), spatial learning, anxiety, behavioural plasticity, brain size

## Abstract

The ways in which challenging environments during development shape the brain and behaviour are increasingly being addressed. To date, studies typically consider only single variables, but the real world is more complex. Many factors simultaneously affect the brain and behaviour, and whether these work independently or interact remains untested. To address this, zebrafish (*Danio rerio*) were reared in a two-by-two design in housing that varied in structural complexity and/or exposure to a stressor. Fish experiencing both complexity (enrichment objects changed over time) and mild stress (daily net chasing) exhibited enhanced learning and were less anxious when tested as juveniles (between 77 and 90 days). Adults tested (aged 1 year) were also less anxious even though fish were kept in standard housing after three months of age (i.e. no chasing or enrichment). Volumetric measures of the brain using magnetic resonance imaging (MRI) showed that complexity alone generated fish with a larger brain, but this increase in size was not seen in fish that experienced both complexity and chasing, or chasing alone. The results highlight the importance of looking at multiple variables simultaneously, and reveal differential effects of complexity and stressful experiences during development of the brain and behaviour.

## 1. Introduction

Since Hebb [1] first described the importance of environmental complexity and its effects on animals, there has been interest in how the environment drives differences in the brain and behaviour. Although the effects of single variables are important (for example, the introduction of enrichment objects into an environment, or varying social interactions in group-housed animals [2]), the natural world is inherently more complex and varies across multiple domains simultaneously. Studies where more than one variable are varied to explore how different factors interact are therefore needed if we are to understand the effects of compound factors and how these affect the development of an animal's brain and behaviour.

Studies on environmental enrichment have described beneficial effects in mammals [3–6], birds [7], reptiles [8] and fishes [9]. Enrichment refers to the addition of physical structures and objects into the environment in which the animals are maintained. Adding enrichment into the housing environment can influence behaviour, positively affecting spatial learning [2,10–12], increasing exploration [13] and decreasing anxiety [11,14]. However, others have failed to find differences, indicating that enrichment does not always have consistent effects [14,15].

Enrichment has been shown to enhance neurogenesis in mammals. The majority of rodent studies have focused on changes occurring within the hippocampus, an area of the brain involved in learning and memory [16]. But again, some studies report beneficial effects on learning [17–19], whereas others find few or no effects [11,20,21].

More recently, zebrafish have been used in studies of enrichment on brain and behaviour, and groups of zebrafish reared with enrichment have been shown to have superior spatial skills compared with fish kept in structurally simple environments [22]. Locomotor behaviour is also altered after only one week of exposure to enrichment [23]. Together, these studies suggest that exposure to enrichment promotes a number of changes that influence the behaviour of zebrafish.

As with rodents, manipulation of the rearing environment can induce changes in the fish brain. Adding a stone substrate in the rearing tanks of developing steelhead salmon (*Oncorhynchus mykiss*) affected the size of the cerebellum [24], and rearing wild-type coho salmon (*Oncorhynchus kisutch*) in a near-natural stream led to larger telencephala [25]. However, coho salmon reared without enrichment developed larger optic tecta and bigger brains, suggesting that brain growth in relation to the environment is not always predictable.

Experience with enrichment may influence neurogenesis because enrichment acts as a kind of stressor, leading to stress inoculation (sometimes referred to as hormesis), but many other forms of stressor also affect changes in brain plasticity. In rodents, different stressors impair neurogenesis (prenatal stress [26], social stress [27,28], physical restraint [29], constant light [30]), compromising cognition [26,30]. But not all stress is bad, and mild stress can enhance cognitive ability; rats given a low level of corticosterone have increased hippocampal cell proliferation and improved spatial learning [31]. Although less is known in fish, chronic stress does negatively affect neurogenesis (cortisol injection [32], social stress [33]).

To investigate the combined effects of enrichment (via environmental complexity) and a daily chasing stressor, zebrafish were reared under different conditions just after hatching. Chasing with a dip net to generate an escape response in the fish was used as a mild, daily stressor. To provide experience of complexity in the environment different structures and objects were added to the tanks to act as enrichment. A two-by-two design was used such that some fish experienced chasing only, others experienced enrichment only, some experienced both, and a final group experienced neither chasing nor enrichment. Fish were reared under these conditions before being tested in two kinds of behavioural assay. First, anxiety was assessed using a novel tank diving test [34]. Fish were screened both as juveniles and as adults to test if effects persisted into adulthood. Second, juvenile zebrafish learning was assessed in a maze to test for effects on cognition. Finally, magnetic resonance imaging (MRI) was used to investigate brain development. Unlike histology or photography, MRI provides a three-dimensional representation of the intact brain and presents a more accurate way to obtain volume measurements.

Previous research has demonstrated that exposure to single factors such as enrichment, or a different form of mild stressor, can generate improved cognition and increased neurogenesis. We predicted that a combination of enrichment and chasing would promote superior learning, relatively low levels of anxiety and a relatively larger telencephalon when compared with fish exposed to just enrichment or chasing, or with control fish reared without experience of either.

## 2. Material and methods

### (a) Treatments

Newly hatched zebrafish fry from four wild-type (EkkWill) pairings were raised until 25 days of age and then evenly distributed across 24 treatment tanks (35 × 19 × 28 cm) such that each tank contained 12 fish (i.e. three siblings from each cross per tank). Multiple pairings were used to avoid possible effects of non-independence within a single brood. Fish were fed daily with commercial flake food and live brine shrimp. Four treatments were created in a 2 × 2 design: (i) enriched, (ii) enriched + chased, (iii) plain and (iv) plain + chased, with six replicate tanks per treatment. Each tank contained a heater and a biofilter, and was maintained on a 12 L : 12 D cycle at 25 ± 1°C. Three of the tank walls were covered with black plastic to minimize disturbance. Enriched tanks contained two plastic plants, one plastic shelter, gravel substrate and a changeable novel object (white PVC pipe, rock, different coloured plants or a plastic bottle). Enriched tank items were moved weekly, and novel objects were switched in and out of the tanks at this time to vary the environment. The heater and biofilter in the plain tanks remained in fixed positions throughout the experiment. enriched + chased and plain + chased treatments experienced daily chasing with a small dip net. Four figure-of-eight sweeps of the net were made across the top and bottom of the tank. The order in which the tanks were chased was changed each day.

After 52 days of treatment exposure, when the fish were 77 days old, a subset of fish were screened to assess anxiety. After 60 days of exposure a second subset of fish were tested in a learning task. After these assays were completed, at 78 days of exposure to the treatments, one fish from each tank was sacrificed for MRI (see below). Throughout these tests, fish from all replicate tanks were evenly selected and no fish were re-used.

All remaining fish were then transferred to standard rearing conditions in tanks with a heater, a biofilter and a water depth of 25 cm, but without enrichment or chasing. As the fish reached sexual maturity, the sexes were separated such that within each treatment males were divided across two tanks and females (or fish that could not reliably be sexed) were in two other tanks (dimensions: 90 × 32 × 30 cm), creating four tanks per treatment. At one year old, adult male fish were screened in the anxiety assay.

### (b) Anxiety assay

The first anxiety assay was performed after 52 days of treatment exposure (*n* = 18 per treatment) when fish were screened in a novel tank diving test [34]. At this stage the zebrafish were immature and could not be sexed. The second anxiety assay was run with males only when the fish were one year old and could be sexed (*n* = 10 males).

The test tank (juveniles: 26 × 15 × 17 cm, water depth 14 cm; adults: 35 × 19 × 28 cm, water depth 24 cm) was covered on three sides with black plastic. A camera facing the uncovered side of the tank recorded behaviour. Filming started as a fish was released into the top 2 cm of the water. Each fish could explore the tank for 5 min. After each trial, a third of the water was exchanged and mixed before a new fish was tested.

Videos were analysed using Etholog v. 2.2.5 [35]. The test tank was visually divided into bottom, middle and top zones using a 3 × 3 cm grid superimposed on the monitor. When first released, all fish swam to the bottom of the tank. Data collection began once a fish reached the bottom (approx. 5 s). A summary of the behaviours measured during testing are listed in the electronic supplementary material, table S1.

### (c) Learning assay

For the maze task, the selected test fish (*n* = 18 per treatment) were housed in a single tank per treatment during these trials. The maze, positioned in the middle of the test tank, consisted of a central arena (length 13 cm × width 8 cm) with exits (5 × 2.5 cm) at each corner. Three exits were ‘false’ and led to dead ends, but one ‘true’ exit led out into the open area of the test tank. The ‘true’ exit was marked with a small black circular piece of plastic (radius 3 mm) attached to the wall.

Trials began with a single fish in a transparent tube (diameter 5 cm) in the maze centre. After 30 s, the fish was released and allowed to explore the maze. Trials lasted 5 min or until the fish found the true exit. Zebrafish are a shoaling species [36], thus fish were trained to find a stimulus school on exiting the maze [37,38]. If a fish did not find the exit within 5 min, it was netted and placed outside of the maze. After each test, water from the centre of the maze was removed and replaced with fresh water. No fish re-entered the maze. Fish were tested once a day for 9 days and each trial was filmed using a camera positioned above the test tank. Etholog v. 2.2.5 was used to quantify the final time to exit the maze as well as the number of false arms entered (i.e. mistakes).

### (d) Magnetic resonance imaging and image post-processing

After the behavioural trials were completed, one untested fish from each replicate treatment tank (*n* = 6 per treatment) was prepared for MRI. Fish were euthanized (buffered 2 g l^−1^ MS-222), standard length measured, then rinsed and fixed in a 5 ml solution of 10% neutral buffered formalin for 72 h. They were then immersed in a 2% Magnevist (Bayer HealthCare) phosphor-buffered solution for one week and stored at 4°C. Magnevist helped to reduce the T1 (26.9 ± 0.4 ms^−1^) and T2 (7.5 ± 0.1 ms^−1^) times of the tissue, therefore permitting faster imaging.

During scanning, specimens were surrounded by a flourinert liquid FC-43 (3 M) with cotton wool to inhibit the movement of the sample in the vial. All specimens were scanned at the High Field MRI Facility at the Pennsylvania State University, in a vertical 14.1 tesla Agilent imaging system using a home-built saddle coil. A standard three-dimensional spin echo sequence with an isotropic resolution of 20 µm comprised a field of view of 10 × 4 × 3 mm and a matrix size of 500 × 200 (0.75 partial Fourier = 150) × 150. With 32 averages and a repetition time of 70 ms (echo time 8.85 ms) the total scan time was 14 h.

Matlab (The Math Works) was used for post-processing. Data were zero-filled by a factor of 2 in each direction resulting in a 10 µm isotropic pixel resolution. Brain volumes were measured using three-dimensional data visualization software (Avizo v. 6.2.1, VSG Inc., Burlington, MA, USA), and manual segmentation was performed by a single person using the ‘Label-field’ segmentation editor in Avizo and verified using neuroanatomical references (e.g. [39]).

**Figure 1.**
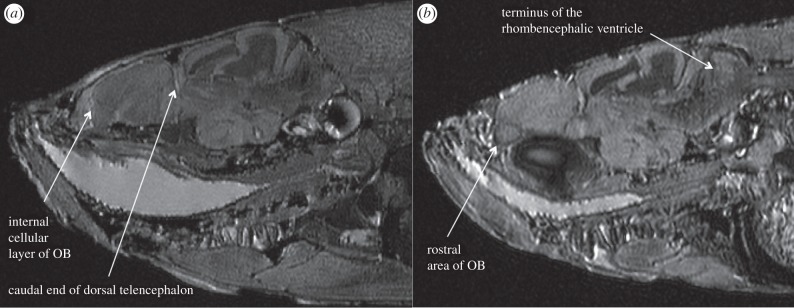
Sagittal magnetic resonance images showing structures used to delineate (*a*) the telencephalon and (*b*) the whole brain. OB, olfactory bulb.

The telencephalon was segmented every 3rd slice (30 µm) in the sagittal perspective and confirmed by inspection in the axial and coronal views. The whole brain was segmented every 6th slice (60 µm) in the axial perspective and again confirmed using the two other orthogonal views. The telencephalon was defined as extending from the terminus of the internal cellular layer of the olfactory bulb caudally to the end of the dorsal telencephalon following Lema *et al*. [40]. We chose to use structures with clear, distinguishable boundaries to define the areas we measured. For this reason, the pre-optic area (POA) was included in all telencephalon volume measurement, and the whole brain was defined as extending from the most rostral area of the olfactory bulb (glomerular layer of the olfactory bulb) to the terminus of the rhombencephalic ventricle caudally. These structures had readily distinguishable boundaries that could be defined in all specimens (figure 1). Volumes were calculated using the ‘Materials–Statistics’ function of Avizo. To decrease measurement error, whole brain and telencephalon volumes were measured three times, and the mean of these was used for statistical analysis.

### (e) Statistical analysis

Data were analysed using general linear models in a 2 × 2 between-factor design. Data were tested for equality of variance and transformed when necessary. Diving variables were compared across treatments with housing (enriched or plain) and stressor (chased or not chased) as independent variables with two levels each. Time spent in the bottom of the tank was compared across treatments, with minute of observation as a fixed-effect variable and individual fish as a random-effect variable. For the learning assay, housing and chasing treatments were considered independent variables with two levels each, and mean latency to exit or mean number of arms visited as dependent factors. A tank effect was not included as fish from each replicate tank were mixed in one holding tank per treatment, and the small size of the juvenile fish prevented us from marking the fish. One fish died 2 days into testing and was excluded from analyses.

There was no effect of enrichment (*F*_1,20_ = 2.54, *p* = 0.12) or chasing (*F*_1,20_ = 0.22, *p* = 0.64) and no interaction (*F*_1,20_ = 0.01, *p* = 0.93) on standard length of the fish. Relative measures of telencephalon size in fish have used both whole brain and standard length to size-standardize results [24,25,41–43]. We analysed the data both ways. There was no effect of enrichment (*F*_1,20_ = 4.59, *p* = 0.06) or chasing (*F*_1,20_ = 0.05, *p* = 0.82) and no interaction (*F*_1,20_ = 1.92, *p* = 0.18) on absolute brain volume. Both regression of telencephalon volume on standard length (*R*^2^ = 0.85, *p* < 0.01) and overall brain volume (*R*^2^ = 0.97, *p* < 0.01) were significant and thus were used to size-standardize the results. A 2 × 2 between-factor design was again used to compare relative telencephalon volume (using standard length or whole brain volume) across treatments. Analysis of telencephalon volume relative to body size indicated a size difference between treatment groups, but this effect was not apparent when relative to whole brain volume. To investigate the possibility that the whole brain and not just the telencephalon was bigger in one of the treatment groups, further analyses were carried out with relative brain volume calculated using standard length as the standardizing measure and then compared across treatments. Analyses were performed in SPSS (v. 21) and significance was tested at *α* = 0.05. Values are quoted as mean ± s.e.m.

## 3. Results

### (a) Anxiety assessment

**Figure 2.**
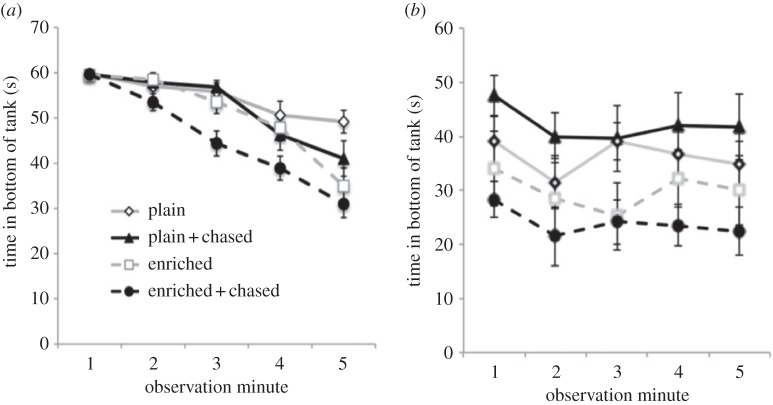
Time spent at the bottom during the novel tank diving test during each minute of the trial. (*a*) There were main effects of both enrichment (*F*_1,68_ = 11.96, *p* < 0.01) and chasing (*F*_1,68_ = 6.17, *p* = 0.02) for fish tested as juveniles, and (*b*) a main effect of enrichment (*F*_1,36_ = 7.96, *p* < 0.01) in fish tested as adults. Mean ± s.e.m.

When juvenile fish were tested, fish reared in plain tanks without chasing spent more time in the bottom of the test tank, showing increased signs of anxiety compared with fish that experienced enrichment and chasing (main effect of enrichment: *F*_1,68_ = 11.96, *p* < 0.01; main effect of chasing: *F*_1,68_ = 6.17, *p* = 0.02; figure 2*a*). There was no overall difference in the rate of movement between treatments (enrichment: *F*_1,68_ = 1.88; *p* = 0.18; chasing: *F*_1,68_ = 2.66; *p* = 0.11), but fish that experienced chasing and enrichment moved into the top of the tank more frequently (main effect of enrichment: *F*_1,68_ = 6.89; *p* = 0.01; main effect of chasing: *F*_1,68_ = 4.88; *p* = 0.03) and stayed at the top for longer (main effect of enrichment: *F*_1,68_ = 3.89; *p* = 0.05; main effect of chasing: *F*_1,68_ = 13.44; *p* < 0.01). Fish that experienced enrichment also spent less time frozen (*F*_1,68_ = 6.71; *p* = 0.01) (for further detail of the novel tank diving assay, see electronic supplementary material, figure S1 and table S2). There were no significant interactions.

When adult zebrafish were tested in the novel tank diving test fish that had previously experienced enriched environments were again less anxious and spent less time at the bottom of the tank (main effect of enrichment *F*_1,36_ = 7.96, *p* < 0.01; figure 2*b*); however, in these adult fish, there was no main effect of chasing (*F*_1,36_ = 0.01, *p* = 0.99).

### (b) Learning assay

**Figure 3.**
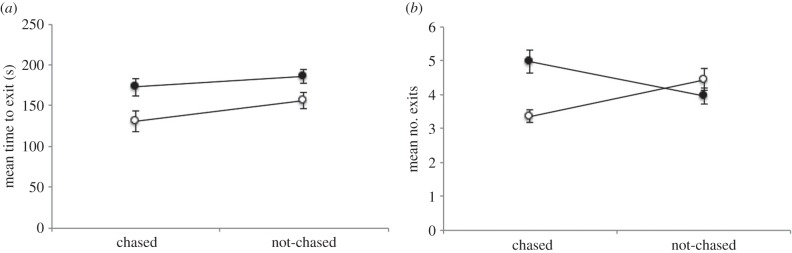
Results of the spatial learning test. (*a*) Mean final time to escape the maze for each treatment group across trials, with a significant effect of both enrichment (*F*_1,8_ = 40.95, *p* < 0.01) and chasing (*F*_1,8_ = 16.44, *p* < 0.01) on time to exit the maze. (*b*) There was a significant interaction between enrichment and chasing (*F*_1,8_ = 12.65, *p* < 0.01) on number of maze arms entered by each treatment group. Plain housing, filled circles; enriched housing, open circles. Mean ± s.e.m.

Fish from the enriched tanks were significantly faster at finding the exit in the maze (main effect of enrichment: *F*_1,8_ = 40.95, *p* < 0.01; figure 3*a*). Similarly, fish that experienced chasing were also faster at locating the maze exit (main effect of chasing: *F*_1,8_ = 16.44, *p* < 0.01; figure 3*a*). There was no interaction (*F*_1,8_ = 0.45, *p* = 0.52).

The number of mistakes made was not affected by the experience of chasing (*F*_1,8_ = 0.11, *p* = 0.92), but the effect of experiencing enrichment was almost significant (*F*_1,8_ = 4.16, *p* = 0.08). There was a significant interaction between enrichment and chasing (*F*_1,8_ = 12.65, *p* < 0.01; figure 3*b*); fish with enrichment made fewer mistakes when exposed to chasing than with no chasing, and fish reared in plain tanks made more mistakes when exposed to chasing than with no chasing (figure 3*b*).

### (c) Brain size

**Figure 4.**
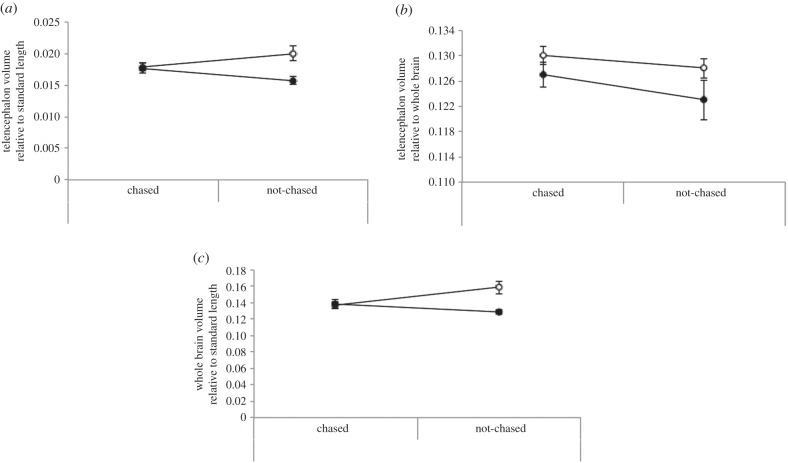
Results of the MRI. (*a*) Volume of the telencephalon relative to whole brain was larger for non-chased fish from enriched housing (*F*_3,23_ = 5.40, *p* < 0.01), (*b*) volume of the telencephalon relative to standard length revealed no significant effects, and (*c*) volume of the whole brain relative to standard length by each treatment group revealed a significant interaction (*F*_1,23_ = 8.91, *p* < 0.01), with fish from the enriched treatment having larger brains compared with plain fish when no chasing occurred. Plain housing, filled circles; enriched housing, open circles. Mean ± s.e.

There was a main effect of enrichment on telencephalon volume when corrected for standard length (*F*_1,20_ = 8.98, *p* < 0.01). There was also an interaction effect (*F*_1,20_ = 7.17, *p* = 0.01; figure 4*a*); enriched fish had a larger telencephalon compared with plain fish when no chasing occurred, but when chased there was no difference in telencephalon volume between the enriched and plain treatments. However, when telencephalon volume was corrected for whole brain size there were no significant results (figure 4*b*). These contrasting results appear to be explained by overall differences in brain volume (corrected for standard length), rather than something specific to the telencephalon; fish from enriched tanks had larger overall brains compared with fish in plain tanks (*F*_1,20_ = 7.16, *p* = 0.02). There was also an interaction effect (*F*_1,20_ = 8.91, *p* < 0.01; figure 4*c*); enriched fish had larger brains (corrected for standard length) compared with plain fish when no chasing occurred, but when chased there was no difference in overall brain size between the enriched and plain treatments.

## 4. Discussion

Exposing young zebrafish to 52 days of enrichment and net chasing enhanced their learning; zebrafish housed with enrichment made fewer mistakes if they were also exposed to chasing with a net, but this result was reversed for zebrafish from plain tanks. Furthermore, zebrafish housed in plain conditions or those that were not chased were slower at finding the maze exit compared with fish housed with enrichment or those that were chased. Fish exposed to enrichment or chasing were also less anxious. Moreover, the effects of rearing environment on anxiety behaviour persisted into adulthood in male fish. Given the use of zebrafish as a model species, rearing methods that promote the development of robust behaviour could promote more consistent individual behaviour patterns [44].

Many mammalian studies have found that exposure to chronic stress impairs learning; however, others have reported no effect, and a debate about whether mild, transient stress facilitates learning has arisen (for reviews, see [45,46]). In this study, fish experiencing chasing were faster to find the maze exit compared with unchased fish, suggesting a superior learning ability. Furthermore, enriched + chased fish made fewer mistakes in the maze, but this effect was not seen in plain + chased fish, indicating that enhanced learning is affected by more than just chasing.

The addition of enrichment to rat cages resulted in decreased stress reactivity and faster habituation to a novel environment [47,48]. In this study, putting fish in the maze is likely to have activated the hypothalamic–pituitary–interrenal (HPI) axis and elevated stress hormone production, especially in the first few trials [49]. The enhanced learning exhibited by enriched + chased fish suggests that early experience in these fish helped them adjust to the challenge of being tested and handled during trials. Results from the diving test support this; both chased and enriched fish were faster at habituating to the novel test environment. Although enriched + chased fish made fewer mistakes in the maze, the opposite was true for plain + chased fish, suggesting that the effects of chasing on learning are affected by exposure to enrichment.

Several factors in fish influence whether exposure to stressors generates an adaptive response (i.e. fight or flight) or maladaptation [50]. External environmental factors such as stressor type, as well as internal processes that switch stress signalling pathways on or off, make it a complex and dynamic system [49]. The novel tank diving test is used in biomedical studies to screen for drugs that influence anxiety disorders or to detect molecular markers of addiction [51]. Fish exposed to enrichment or chasing alone expressed fewer anxiety behaviours, suggesting that both of these factors independently affect how the fish react to novelty.

It is widely accepted that enrichment is beneficial for learning in laboratory rodents. Studies have investigated the effects of either physical or social enrichment on learning [11,48,52–54], with duration of exposure ranging from 21 days [54] to 1 year [52]. Moreover, the beneficial effects of enrichment on learning have been seen at different life stages from development [53] through to adulthood [11,52,54]. We tested exposure over a 52-day period during development to determine whether manipulation of one or two environmental conditions during a critical growth period affected learning. The results indicate that physical enrichment and chasing alone are sufficient to produce changes in learning of juvenile zebrafish; however, the combination of both seems to further enhance this effect.

In terms of changes in the brain, fish from enriched environments had larger brains compared with plain fish. Environmental complexity has been shown to affect the morphology of the telencephalon [55], cerebellum [24,55], olfactory bulbs [55] and optic tectum [25,55] in fish. With the increased capacity of adult neurogenesis in teleost fish it might not be surprising that the environment can exert so many influences on gross brain morphology; however, this also makes it difficult to determine the functional significance of this plasticity. Enrichment increased the size of the cerebellum in steelhead salmon, and this was thought to be associated with differences in locomotion [24]. Whole brain volume was reported to be smaller in group-reared nine-spined sticklebacks (*Pungitius pungitius*) than individually reared fish [56]. These differences were proposed to be a consequence of enhanced sensory structures (such as the optic tectum and olfactory bulbs) because these structures were also found to be smaller in the group-reared fish. In this study, it is possible that fish housed with enrichment were integrating different sensory and motor cues, and the observed increase in brain size was attributed to differences in the size of brain structures that were not measured separately. Moreover, exposure to a mild stressor had differential effects on the brain; when not exposed to chasing, fish reared with enrichment had larger brains compared with fish in plain conditions, but this was not the case for fish exposed to chasing. Interestingly, it was the enriched + chased fish that had enhanced learning and reduced anxiety behaviour, suggesting that brain–behaviour comparisons are not clearly consistent when exposed to enrichment and chasing.

While enrichment is used extensively in mammalian systems, there has been limited application in non-mammalian systems [57]. With regard to captive laboratory fish, zebrafish have shown a preference for an enriched environment [58]. In addition, zebrafish housed with enrichment were faster at finding food rewards in a tank with five chambers [22]. However, Spence *et al*. [22] tested fish in groups, making it difficult to account for individual differences in motivation or differences in social learning. The effects of enrichment and social isolation (used as a short-term stress) have been studied in zebrafish; housing fish with enrichment and isolation increased cell proliferation in the telencephalon. However, there were no learning tests to determine the functional significance of these changes [23]. The results reported here agree with and extend earlier studies; exposing zebrafish to enrichment or chasing alone enhanced spatial learning, but it was a combination of these factors which led to fish making fewer mistakes in the maze, and thus helped to refine learning.

Standard housing conditions for laboratory zebrafish do not include enrichment, and handling is a daily occurrence. Traditionally, laboratory rodents were housed in barren cages, but awareness of welfare has led to social and physical enrichment becoming a standard for rodents [59]. Thus, in order to validate tests for complex behaviours such as anxiety and learning in fish, we need to understand the degree to which previous experience influences these responses [44].

Although the telencephalon is an interesting structure because of its role in learning and modulating stress, the only differences we observed were in overall brain size. While many studies report brain structures relative to a measure of body length, our results demonstrate that if other brain structures are used to size-standardize measurements the outcome can change. Marchetti & Nevitt [41] found differences in the telencephalon and optic tectum relative to standard length in wild versus hatchery reared rainbow trout. Similarly, Burns *et al*. [43] found laboratory-reared guppies had smaller telencephalon and optic tecta relative to standard length compared with wild guppies. In both these studies, it would be interesting to know what the results would have shown had overall brain size been used as the relative measure. Furthermore, most methodologies for measuring substructure volume within the fish brain have followed standard histological techniques [24,42] or measurements taken from photographs [25,41,43]. But distortion of the tissue during histology or measurement error when using photographs can make these methods unreliable. MRI can quantify detailed neuroanatomical information of intact brains in many species [60], and is an effective tool for visualizing the zebrafish brain [61]. While MRI in this study allowed a more accurate measure of brain volume, there were still issues related to image resolution; for example, to ensure that the same structures were consistently measured across fish, we included the POA in the telencephalon measures. Given that major nuclei of the HPI axis are found in this area, it is possible that stress-related aspects of the chasing treatments may have increased the POA volume through increased activation of the HPI axis. This could have masked possible effects on the volume of the telencephalon alone. In future studies, higher-resolution images would allow the telencephalon and POA to be measured separately. A further reason why the ratios between the telencephalon and whole brain measures were not significant may be because of co-regulation between different brain structures such as the cerebellum (similar to observations reported in [55]).

This study emphasizes that zebrafish housed with enrichment or exposed to regular chasing develop reduced anxiety reactivity and have superior learning. In addition, fish performed more accurately in a maze when housed with a combination of enrichment and chasing, but this effect was reversed in fish from plain tanks, suggesting that enrichment is important in enhancing learning. Given the growing use of zebrafish as models for neurobehavioural research, there is a need to understand how different housing and rearing methods influence both brain and behaviour [44]. The results we report here suggest these effects may not be straight forward, particularly when different kinds of rearing experience are combined.

## Supplementary Material

Differences in behaviours between juveniles from different treatments in the novel tank diving test. (a) Latency to top, (b) Entries in top/bottom, (c) Time in top and (d) Log10 Average top entry duration. Summary of behaviours measured for juvenile fish in the novel tank diving test. Adapted from Cachat et al. [34]. Comparison of behaviours in juvenile fish during the novel tank diving test.

## Data accessibility

All raw data datasets associated with this study are lodged with the Dryad database, file: DATASET_DePasquale. Cairsty *et al*. http://dx.doi.org/10.5061/dryad.66216.
