# Supplementary material for: The influence of complex and threatening environments in early life on brain size and behaviour
Source: Proc Biol Sci. 2016 Jan 27;283(1823):20152564. doi: 10.1098/rspb.2015.2564 (PMC4795028; doi:10.1098/rspb.2015.2564)
Supplement: Differences in behaviours between juveniles from different treatments in the novel tank diving test. (a) Latency to top, (b) Entries in top/bottom, (c) Time in top and (d) Log10 Average top entry duration. Summary of behaviours measured for juvenile fish in the novel tank diving test. Adapted from C [file rspb20152564supp1.pdf]

Figure 1 SM.

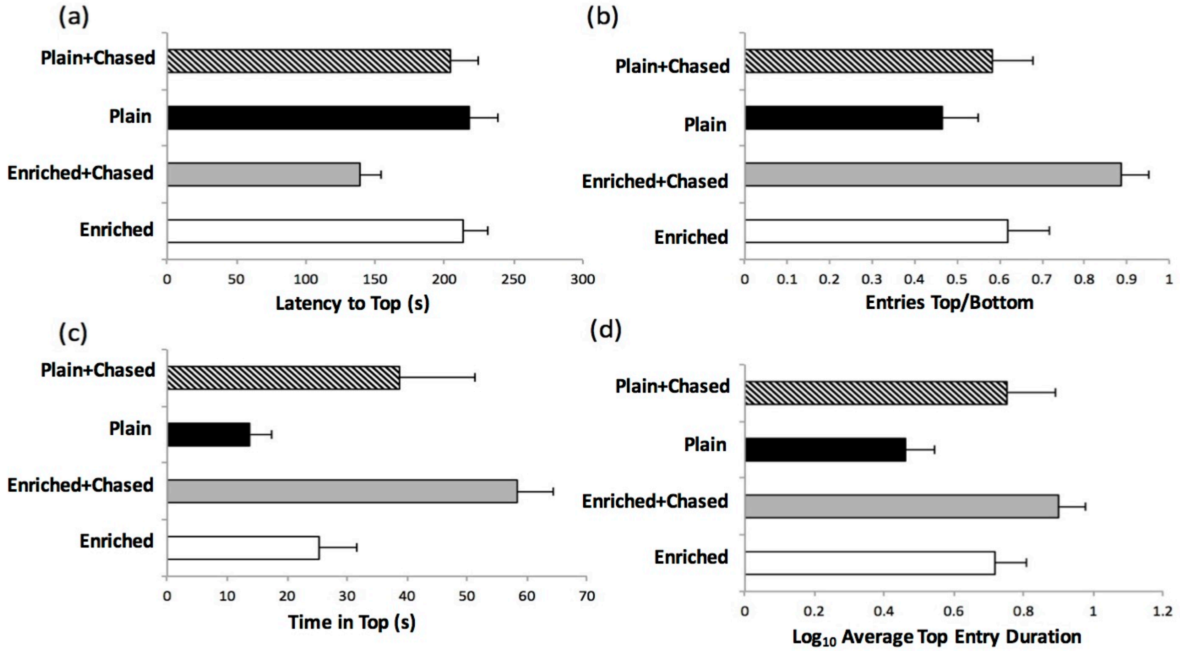

Differences in behaviours between juveniles from different treatments in the novel tank diving test. (a) Latency to top, (b) Entries in top/bottom, (c) Time in top and (d) Log10 Average top entry duration.

Table 1 SM: Summary of behaviours measured for juvenile fish in the novel tank diving test. Adapted from Cachat et al. [34].

**Behaviour (units)**	**Increasing value indicates**
Latency to enter the top (s)	Lower anxiety levels
Ratio of entries to top/bottom	Lower anxiety levels
Time spent in top (s)	Lower anxiety levels
Average top entry duration (s) (time spent in the top divided by the number of entries to the top)	Lower anxiety levels
Freezing duration (s)	Higher anxiety levels
Movement rate (number of grid lines crossed/min)	Lower anxiety levels

Table 2 SM: Comparison of behaviours in juvenile fish during the novel tank diving test.

* Indicates significant difference.

	Enrichment		Chasing	
	**Mean ± SE (Enriched)**<br>**Mean ± SE (Plain)**	**F-value; P-value**	**Mean ± SE (Chasing)**<br>**Mean ± SE (No-Chasing)**	**F-value; P-value**
**Latency to enter top (s)**	176.33 ± 13.15<br>211.16 ± 14.26	$F_{1,68}$ = 3.51; P = 0.06	215.73 ± 13.49<br>171.76 ± 13.57	$F_{1,68}$ = 5.60; P = 0.02*
**Number of entries top/bottom**	0.75 ± 0.06<br>0.52 ± 0.06	$F_{1,68}$ = 6.89; P = 0.01*	0.54 ± 0.07<br>0.73 ± 0.06	$F_{1,68}$ = 4.88; P = 0.03*
**Time in top (s)**	41.83 ± 5.14<br>26.22 ± 6.84	$F_{1,68}$ = 3.89; P = 0.05*	19.51 ± 4.77<br>48.55 ± 6.11	$F_{1,68}$ = 13.44; P < 0.01*
**Top entry duration (s)**	7.81 ± 1.49<br>10.05 ± 5.92	$F_{1,68}$ = 4.00; P = 0.05*	4.35 ± 0.63<br>13.51 ± 5.98	$F_{1,68}$ = 5.48; P = 0.05*
**Freezing duration (s)**	48.57 ± 8.86<br>92.99 ± 14.74	$F_{1,68}$ = 6.71; P = 0.01*	83.81 ± 11.73<br>57.75 ± 13.29	$F_{1,68}$ = 2.31; P = 0.13
**Movement rate (lines/min)**	35.06 ± 1.87<br>30.82 ± 2.50	$F_{1,68}$ = 1.88; P = 0.18	30.41 ± 2.25<br>35.46 ± 2.14	$F_{1,68}$ = 2.66; P = 0.11
